# *Mycobacterium* Growth Inhibition Assay of Human Alveolar Macrophages as a Correlate of Immune Protection Following Mycobacterium bovis Bacille Calmette–Guérin Vaccination

**DOI:** 10.3389/fimmu.2018.01708

**Published:** 2018-07-24

**Authors:** Juliane Radloff, Jan Heyckendorf, Lize van der Merwe, Patricia Sanchez Carballo, Norbert Reiling, Elvira Richter, Christoph Lange, Barbara Kalsdorf

**Affiliations:** ^1^Division of Clinical Infectious Diseases, Research Center Borstel, Borstel, Germany; ^2^German Center for Infection Research (DZIF), Hamburg, Germany; ^3^International Health/Infectious Diseases, University of Lübeck, Lübeck, Germany; ^4^Division of Microbial Interface Biology, Research Center Borstel, Borstel, Germany; ^5^National Reference Center for Mycobacteria, Research Center Borstel, Borstel, Germany; ^6^Department of Medicine, Karolinska Institute, Stockholm, Sweden

**Keywords:** tuberculosis, vaccine, mycobacterial growth inhibition assay, Bacille Calmette–Guérin, vitamin D, interferon-γ release assay immune response

## Abstract

**Background:**

In order to eliminate tuberculosis (TB), an effective vaccine is urgently needed to prevent infection with *Mycobacterium tuberculosis*. A key obstacle for the development of novel TB vaccines is the lack of surrogate markers for immune protection against *M. tuberculosis*.

**Methods:**

We investigated growth rates of *M. tuberculosis* in the mycobacterial growth inhibition assay (MGIA) as a marker for mycobacterial growth control of human bronchoalveolar lavage (BALC) and peripheral blood mononuclear cells (PBMC) before and after vaccination with *Mycobacterium bovis* Bacille Calmette–Guérin (BCG) of healthy adult volunteers.

**Results:**

Vaccination induced a positive response (*p* < 0.001) to purified protein derivate (PPD) in 58.8% of the individuals in an interferon-γ release assay-ELISpot. Intraindividual evaluation of the MGIA growth rates before and after *M. bovis* BCG-vaccination revealed no significant difference in time to culture positivity before and after vaccination in BALC (*p* = 0.604) and PBMC (*p* = 0.199). The magnitude of the PPD-response induced by *M. bovis* BCG-vaccination did not correlate with growth control in BALC and PBMC (correlation = 0.468, 95% CI: −0.016 to 0.775).

**Conclusion:**

In conclusion, *M. bovis* BCG-vaccination-induced mycobacterial-specific cytokine immune response does not result in functional immune control against *M. tuberculosis* in the MGIA.

## Introduction

Tuberculosis (TB) is a leading cause of morbidity and mortality worldwide (1). In the year 2016, the World Health Organization reported 10.4 million new cases of TB. Elimination of TB appears to be an unrealistic goal in the near future unless TB prevention can be dramatically improved. Prevention of TB can be achieved by vigorous infection control measures, treatment of individuals that are latently infected with *Mycobacterium tuberculosis* (LTBI) and most effectively by a preventive vaccine. Almost 100 years ago, Albert Calmette and Camille Guérin developed a vaccine based on attenuated *Mycobacterium bovis* [Bacillus Calmette-Guérin (BCG)], which is still the only available anti-TB vaccine in clinical use today (2, 3). Although *M. bovis* BCG is among the most commonly used of all vaccines worldwide, its effect is largely on the attenuation of severe forms of the disease in children and it does not prevent TB in adults (4, 5).

In view of this important limitation, there is an urgent need for the development of a novel TB vaccine, with the property to prevent active TB in adults and children (6–8). None of the candidates have shown a significant protective effect against TB infection (7–11). The main obstacle to validate a new vaccine candidate’s ability to protect against *M. tuberculosis* infection is the lack of a robust correlate of protection (12).

The *in vitro* mycobacterial growth inhibition assay (MGIA) appears to be a promising marker to detect protective immunity of vaccine-induced cells toward *M. tuberculosis* (13–17). Improved immune control of mycobacterial growth was demonstrated following primary *M. bovis* BCG-vaccination when compared to individuals who had been *M. bovis* BCG-vaccinated as a child and revaccinated (15). In another human challenge trial, improved immune control was reported in individuals with past *M. bovis* BCG-vaccination, but no additional protective effect was seen after MVA85A-vaccination (16). And H56:CAF01-vaccination revealed an enhanced immune-control in the MGIA corresponding to *in vivo* protection in mice (17). So far, the MGIA has been evaluated in the following cell types and species: splenocytes (17–19) and bone-marrow-derived macrophages in mice (20), whole blood of Rhesus macaques (21), whole blood cells, frozen and fresh peripheral blood mononuclear cells (PBMC) (15, 16, 22, 23), and antigen-expanded T-cells (24) in humans.

In order to test the MGIA as a pulmonary correlate of immune protection from mycobacterial infection, we recruited *M. bovis* BCG-naïve healthy adult volunteers and analyzed mycobacteria-specific immune responses and growth of *M. tuberculosis* in an *ex vivo* growth inhibition model in bronchoalveolar lavage cells (BALCs) and PBMC before and after vaccination with *M. bovis* BCG.

## Materials and Methods

### Study Design

Healthy *M. bovis* BCG-vaccine-naïve subjects underwent phlebotomy and bronchoscopy before and 8 weeks after intracutaneously administered *M. bovis* BCG-vaccination. At both time points, BALC and PBMC were *in vitro* infected with *M. tuberculosis* (H37Rv) and cultured for 96 h. *M. tuberculosis* growth rate was determined by the time to culture positivity (TTP) in the MGIA in liquid cultures [*Mycobacterium* growth indicator tube (MGIT)], Becton, Dickinson and Company (BD), Franklin Lakes, NJ, USA, and colony forming units (CFU) on solid cultures (Middlebrook 7H10, BD). BALC and PBMC were characterized by flow cytometry for CD3, CD4, CD8, Granulysin, Granzyme B, and Perforin expression. T-cell immune responses of PBMC were further analyzed in an interferon-γ release assay (IGRA) enzyme-linked immunospot assay (ELISpot).

### Study Approval

This study complied with the declaration of Helsinki (2008) and ethics approval was obtained from the Ethics Committee of the University of Lübeck (14-091). Reporting follows the “Standards for Reporting of Diagnostic Accuracy” (STARD).

### Inclusion and Exclusion Criteria

Participants had no history of TB or *M. bovis* BCG-vaccination and negative IGRA results in PBMC to exclude LTBI at the beginning of the study.

**Figure 1:**
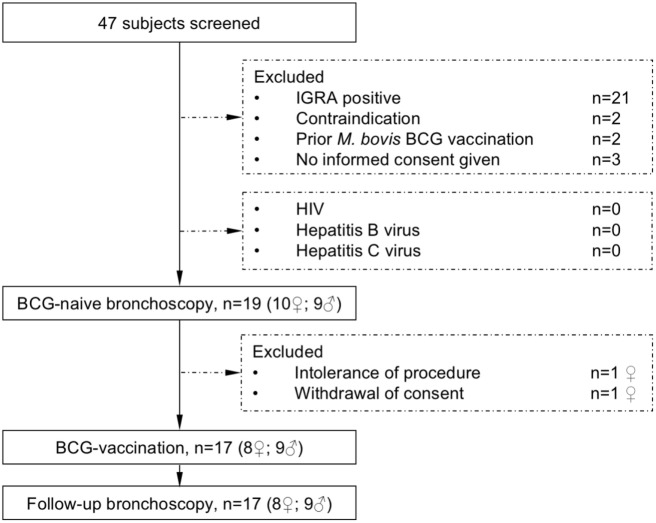
The study flow diagram describes participant screening and study interventions. The interferon-γ release assay (IGRA) was performed to exclude previous contact to mycobacteria. All subjects were healthy and not infected with HIV (human immunodeficiency virus) or hepatitis B or C virus before receiving *Mycobacterium bovis* Bacille Calmette–Guérin (BCG) vaccination.

To reduce potential adverse events of *M. bovis* BCG-vaccination, volunteers were investigated for their health-status and immune-competence by testing for HIV, Hepatitis B or C-infection, analysis of the CD4/CD8 T-cell quotient and the differential blood cell-count and immunoglobulin status by serum electrophoresis. Pregnancy was excluded in female participants. Before bronchoscopy, the differential blood cell-count and coagulation parameters were analyzed repeatedly and symptom screen for any on-going viral or bacterial infection and physical examination were performed. The study flow diagram is shown in Figure 1.

### Cell Sampling and Preparation

Flexible bronchoscopy including bronchoalveolar lavage (BAL) of the middle lobe was performed with 300 mL saline by a pulmonologist according to German national recommendations (25). Sedation was achieved by the application of intravenous midazolam and propofol.

Bronchoalveolar lavage was processed immediately to isolate the BALC as previously described (26, 27). In brief, after centrifugation (10 min, 578 *g*, 4°C) and washing steps, cells were counted and adjusted to 10 Mio/mL in cell culture medium (Rosewell Park Memorial Institute; RPMI with 5 µg/mL Amphotericin B, 100 U/mL Penicillin G and 5% human serum—Sigma-Aldrich Corp., St. Louis, MO, USA). Lavage differential cell counts were performed on cytocentrifuged preparations stained by “hemacolor rapid staining of blood smear” (May-Grünwald-Giemsa; Merck, Germany). Thousand cells per sample were counted.

Peripheral blood mononuclear cells were isolated from venous lithium-heparin blood by ficoll density centrifugation, washed, counted, and adjusted for 10 Mio/mL in cell culture medium as previously described (28, 29).

### Interferon-γ Release Assay

As antigen-specific interferon gamma release assay, an ELISpot (T-Spot.TB^®^ test, Oxford Immunotec, Abingdon, UK) was performed on PBMC as recommended for blood according to the manufacturer’s recommendation (30). 200,000 PBMCs were cultured in RPMI medium containing 1% penicillin–streptomycin and 5%-fetal-calf-serum (FCS) at 37°C on a precoated 96-well T-spot.TB^®^ test plate (Oxford Immunotec). Negative controls were left unstimulated, positive controls were stimulated with anti-CD3-antibody (10 ng/mL, Beckmann Coulter, Brea, CA, USA). Purified protein derivative (PPD) (10 µg/mL, Statens Serum Institute, Copenhagen, Denmark) was utilized to detect cellular immune-responses toward *M. tuberculosis, M. bovis* BCG, or non-tuberculous mycobacteria (31, 32). Spot forming cells (SCF) were counted after 18–24 h incubation.

ELISpot assay results were considered conclusive if the number of SFCs in the positive control well was more than 20 SFCs after subtracting the number of spots in the negative control well and had less than twice the number of spots of the negative control well, positive if more than five spots were counted in the ESAT-6 or CFP-10 well after subtracting the number of the SFC in the negative control and if the total number of SFC was at least twice the number of the negative control, and negative in any other case.

### *M. tuberculosis* Inoculum

*Mycobacterium tuberculosis* H37Rv strain ATCC 27294 was grown in Middlebrook 7H9 broth (BD) supplemented with Middlebrook OADC enrichment medium consisting of 600 µg/mL oleic acid, 50 mg/mL bovine albumin, 20 mg/mL dextrose, and 30 µg/mL catalase (OADC, Life Technologies, Gaithersburg, MI, USA), as well as 0.002% glycerol, and 0.05% tyloxapol. Midlog phase cultures were harvested, aliquoted, and frozen at −80°C.

A new vial was thawed for every experiment and dilution series of the bacterial suspension were prepared in MGIA assay medium for *M. tuberculosis*. Viable *M. tuberculosis* counts were determined by plating serial dilutions of the cultures on Middlebrook 7H10 agar plates. This enabled us to calculate the absolute number of CFU as inoculum per experiment. Furthermore, the infection inoculum was directly suspended into MGIT and tubes were placed in a BACTEC 960 instrument until tubes were detected positive. For each test series, the TTP and the concentration of *M. tuberculosis* measured by CFU were paired and the coefficient of variation was calculated between the experiments.

To determine the necessary bacterial inoculum to reach 156 h in TTP, which fits the midlog phase of the mycobacterial growth and is said to be the widest window of growth inhibition (19), several pre-experiments with increasing concentrations of *M. tuberculosis* and different cell numbers were performed (Figure S1 in Supplementary Material).

### Mycobacterial Growth Inhibition Assay

One million of each of PBMC or BALC were infected with the dosage multiplicity of infection (MOI) of 0.058 (58,000 *M. tuberculosis* in CFU with 1 Mio cells) per well on a 24-well plate (Nunclon™ Delta surface, Apogent, Roskilde, Denmark) in 600 µL RPMI without HEPES and 5% (0.05 mL/mL) human serum. Since lavage is not physiologically sterile (33), all cells were cultured in the presence of antibiotics (100 U/mL penicillin G, and 5 µg/mL amphotericin B, both Biochrom AG, Berlin, Germany). As control group, *M. tuberculosis* was cultured for 96 h in cell culture medium without cells. Infection experiments in the MGIA were performed in duplicates, plates were incubated at 37°C and 5% CO_2_ atmosphere. After 96 h, the mycobacterial growth in PBMC or BALC was stopped by cell lysis with hypotonic aqua destillata. The mycobacterial suspension was transferred into the two different bacterial growth systems: MGIT and agar plates.

### Time to Culture Positivity (TTP) in MGIT

After the infection assay, the MGIA-lysate was transferred to the MGIT system, containing liquid 7H9 medium (Middlebrook Bouillon, BD, Franklin Lakes, NJ, USA) with OADC Supplement and PANTA (polymyxin B 400 U/mL, amphotericin B 40 µg/mL, nalidixic 160 µg/mL, trimethoprim 40 µg/mL, and 40 µg Azlocillin/mL). The MGIT device recorded electronically the start time of incubation and the time at which the culture turned positive. Each infection inoculum from day 0 was processed in the same manner.

### Colony Forming Units (CFU) on Middlebrook 7H10 Agar Plates

Three serial decimal dilution steps of the MGIA-lysate of day 4 and the infection inoculum on day 0 were plated in triplicates on Middlebrook 7H10 agar plates to determine mycobacterial growth in CFU. Plates were incubated airtight with 5% CO_2_ for 2 weeks. CFUs were counted manually.

### MGIA With *In Vitro* Supplementation of Vitamin D

In a subset of experiments, the MGIA with PBMC or BAL was performed in the presence of 30 µmol/L vitamin D_3_ (1α,25(OH)_2_-cholecalciferol solved in dimethyl sulfoxide), which was added to the plain mycobacterial well, as well as to the infection wells with PBMC plus *M. tuberculosis* or BALC plus *M. tuberculosis*.

### Flow Cytometry

For phenotypic analysis, freshly isolated PBMC and BALC were stained (20 min, 4°C, light-protection) with the following surface antibodies (all BioLegend; SanDiego; CA, USA): anti-CD3 PerCP Cy5.5 (Clone UCHT1), anti-CD4 Brilliant Violet 510 (Clone OKT4), anti-CD8a APC-Cy7 (Clone RPA-T8). Afterward, cells were permeabilized with 250 µL Cytofix/Cytoperm (BD) for 30 min at 20°C, washed twice, centrifuged (7 min, 912 *g*, 4°C), and decanted. Intracellular staining was performed with anti-Granzyme B Pacific blue (Clone GB11), anti-Perforin PE (Clone dG9), and anti-Granulysin Alexa Fluor 647 (Clone DH2) for 30 min at 4°C. Acquisition was performed on a FACSCanto II^®^ flow cytometer (BD). Gating strategy is shown in Figure S2 in Supplementary Material. Data were analyzed with FlowJo software version 10.2 (TreeStar, Ashland, TX, USA).

### Statistical Analysis

General linear mixed-effects models were used to describe and graph the estimated effects of all predictors (except eosinophils and PPD outliners, described later) and provided *p*-values for differences between groups, while adjusting for the possible confounding effects of the other predictors. Because multiple observations for each individual in the study were present, the models were corrected for the correlation between observations on the same individual by including the individual identities in the model as random effects. The predictors were several groupings (such as *M. bovis* BCG vaccination: after/before and vitamin D: no/yes), and the numerical predictor, dilution of the outcome. The CFU counts and the fluorescence data were log-transformed to approximate normality for all analyses. As each outcome was modeled once only, all estimates, including *p*-values, for a specific outcome, will be adjusted for each other. McNemar tests were used to estimate the *p*-values for differences in eosinophils and PPD outliners in Table 1. Notched boxplots are used to illustrate the distribution of outcomes in groups. The notches represent approximate 95% confidence intervals for the medians. If the notches do not overlap, the medians of the groups probably differ significantly, and *vice versa*. All statistics was done in base R: a language and environment for statistical computing, and R packages *lmer* and *lmerTest* (https://www.r-project.org/).

**Table 1:** Cell characteristics and cytokine immune responses.

	Before vaccination *n* = 19		After vaccination *n* = 17		*p*-Value
Bronchoalveolar lavage cells (BALC)/100 ml bronchoalveolar lavage %, median (IQR)	6.7	(4.9–11.3)	10.4	(4.4–15.8)	0.1690
BALC differentiation
Macrophages %, median (IQR)	89.0	(86.5–93.0)	95.0	(91.0–97.0)	0.0063
Lymphocytes %, median (IQR)	8.0	(6.5–11.5)	3.0	(2.0–7.0)	0.0011
Neutrophiles %, median (IQR)	2.0	(1.0–3.0)	2.0	(1.0–2.0)	0.4530
Eosinophiles, *n* (%)	0	(0.0%)	1	(5.9%)	0.4430
Purified protein derivate (PPD) positive response in peripheral blood mononuclear cell (PBMC), *n* (%)	0	(0.0%)	10	(58.8%)	0.0020

*Cell frequencies and cell differentials in BALC and PPD-induced interferon-γ release assay (IGRA) immune responses in PBMC before and after *Mycobacterium bovis* Bacille Calmette–Guérin vaccination. Lavage differential cell count was carried out manually on 1,000 cells by microscope. The table shows the median (IQR) for the first five outcomes and count (%) for eosinophils and PPD-positive response, stratified by time point. The *p*-values are given for the difference between after and before*.

## Results

### Demographics and Patient Characteristics

**Figure 2:**
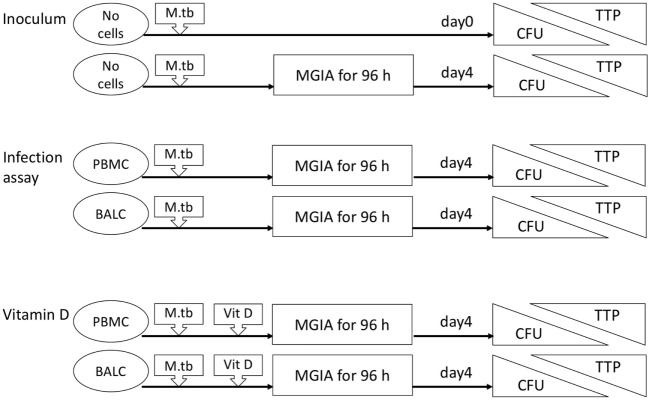
Flow diagram of the different sets of experiments. The inoculum experiment contained no cells but only *Mycobacterium tuberculosis* H37Rv in dilution. Colony forming units (CFU) and time to culture positivity (TTP) were measured as readings in all mycobacterial growth inhibition assays (MGIA) after 96 h and also immediately in the inoculum experiments. Peripheral blood mononuclear cells (PBMC), bronchoalveolar lavage cells, respectively, were added in the infection assay with *M. tuberculosis*. In addition to these cells, vitamin D (Vit D) was supplemented in the vitamin D experiments.

Nineteen healthy volunteers (nine male, ten female) with a median age of 25 years (range 19–56 years) were recruited for this intervention study. After the first bronchoscopy, one study participant withdrew her consent and one participant was excluded due to hypotension side effects following the first bronchoscopy. Seventeen participants were vaccinated with *M. bovis* BCG and received a follow-up bronchoscopy 8 weeks after vaccination (median 56 days, range 54–70 days, Figure 1). Figure 2 gives an overview for the different sets of experiments performed.

### Cell Differentiation and IGRA-Immune Responses

Differentiation in the cell composition of BAL did not change before and after vaccination*. M. bovis* BCG-vaccination induced positive blood IGRA immune responses against PPD in 10/17 participants (58.8%, Table 1).

### *M. tuberculosis* Inoculum

**Figure 3:**
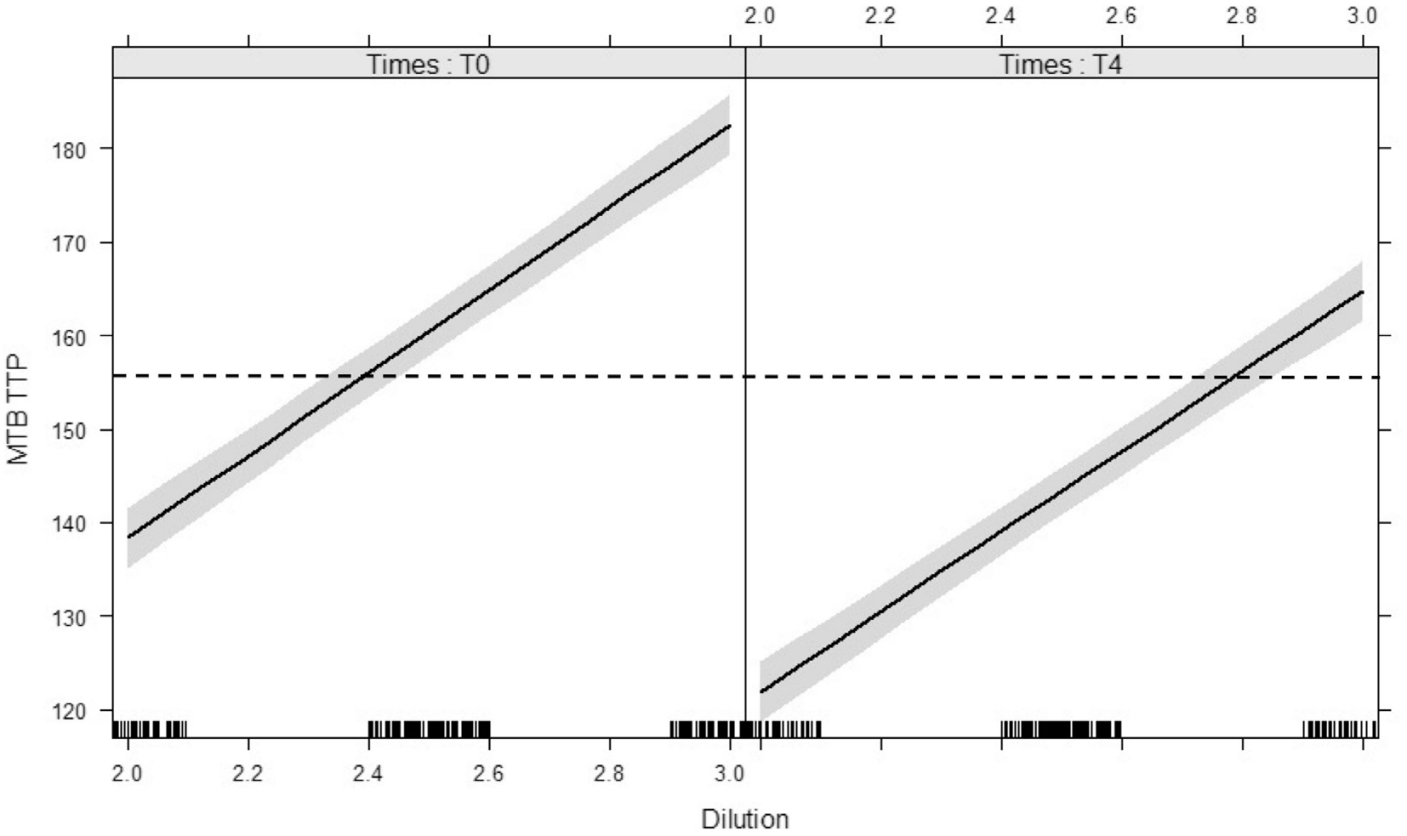
Modeled lines between dilution of *Mycobacterium tuberculosis* H37Rv inocula and the time to culture positivity (TTP) on day 0 and day 4. The time target of 156 h is marked with a dotted line, the 95% confidence bands are gray. There is no interaction between time (day 0 vs. day 4) and dilution on TTP. Both main effects (slope and intercept) are significant. The modeled lines with their 95% confidence bands illustrates that the slopes of the lines are significant but not different and the intercepts differ significantly.

Reproducibility of the inoculum was illustrated by TTP dilution curves repeated for each experiment on day 0 and day 4 (Figure 3): each dilution unit step resulted in an estimated Δ 43.5 h increase in TTP (95% CI: 40.7–46.2 h, *p* < 0.0001). The inoculum at day 4 represented the growth of the *M. tuberculosis* inoculum, after 96 h MGIA in cell culture medium without cells, and was used as blank control. Due to the decreased growth of *M. tuberculosis* in RPMI without cells, TTP values were on average 17.1 h lower in the inoculum on day 4 than on day 0 (95% CI: 15 to 19.1 h, *p* < 0.0001).

### Infection Assays

**Figure 4:**
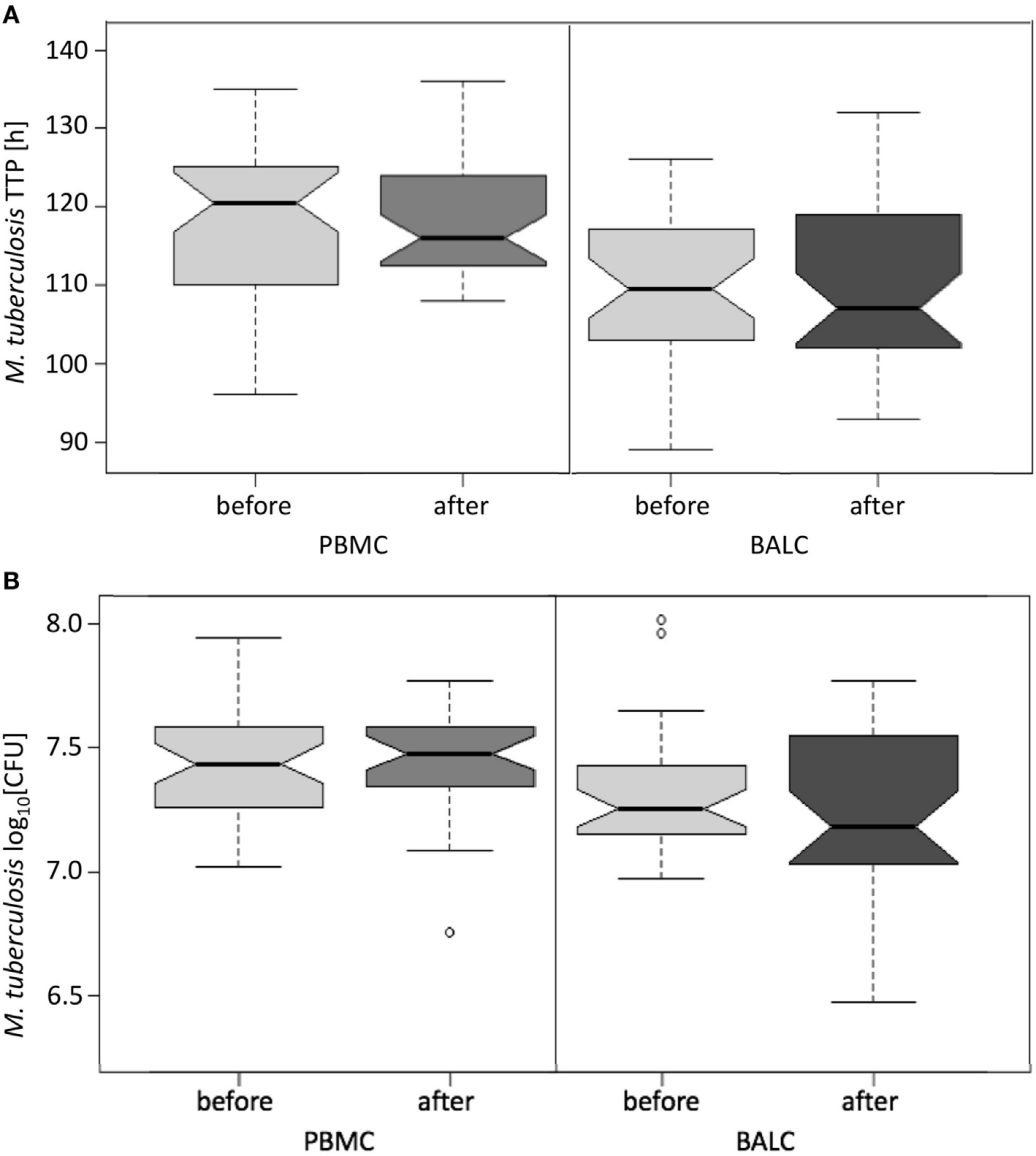
Notched boxplots of the result of *Mycobacterium bovis* Bacille Calmette–Guérin (BCG) vaccination on healthy human adults in peripheral blood mononuclear cells (PBMC) and Bronchoalveolar lavage cells (BALC) in an *in vitro* infection assay with *Mycobacterium tuberculosis* H37Rv. *M. tuberculosis* growth was measured **(A)** by time to culture positivity (TTP) or **(B)** colony forming units (CFU).

The growth of *M. tuberculosis* was tested in PBMC and BALC, before and after *M. bovis* BCG-vaccination (Figure 4A). Comparing the capability of cells to restrict *M. tuberculosis-*growth before and after vaccination, no significant difference in TTP in PBMC (after–before = 2.2 h, *p* = 0.1990) nor in BALC (after–before = 0.9 h, *p* = 0.6040) was shown. Irrespective of *M. bovis* BCG-vaccination, growth (TTP) in PBMC was an estimated 7.6 h longer than in BALC before vaccination (95% CI: 4.3–10.9 h; *p* < 0.0001) and 9.0 h longer after vaccination (95% CI: 5.6–12.4 h; *p* < 0.0001).

**Figure 5:**
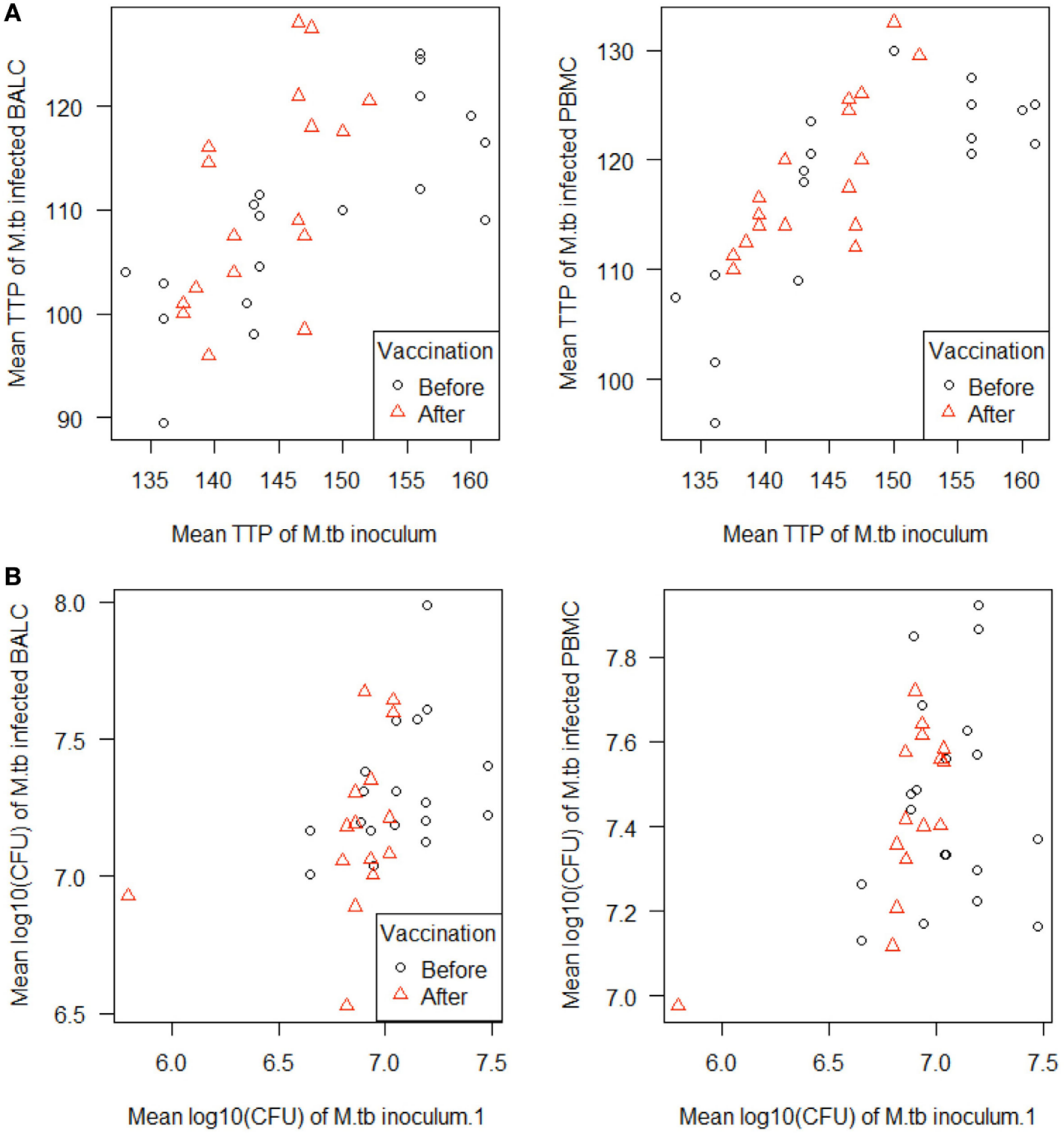
Scatterplots of the *in vitro Mycobacterium tuberculosis* H37Rv-infected cells [peripheral blood mononuclear cells (PBMC) and bronchoalveolar lavage cells (BALC), respectively] plotted against the *M. tuberculosis* inoculum. *M. tuberculosis* growth was measured as **(A)** time to culture positivity (TTP) or **(B)** colony forming units (CFU) at both time points before (γ) and after vaccination (Δ).

In PBMC, the CFU counts did not differ between before and after *M. bovis* BCG-vaccination (estimate 0.01 log_10_, *p* = 0.7512) (Figure 4B). After *M. bovis* BCG-vaccination, the BALC had an estimated 0.1 lower log_10_ CFU count (95% CI: 0 to 0.2 h *p* = 0.0231) than BALC before vaccination. But this effect had to be relativized by the deviation of the infection inoculum, when each TTP of the *M. tuberculosis* inoculum per experiment was plotted against the corresponding TTP of the *M. tuberculosis* infected cell type (Figure 5A shows TTP data, Figure 5B CFU data, respectively). *M. tuberculosis* growth measured as TTP or CFU was not affected by BCG-vaccination.

### Vitamin D Supplementation

**Figure 6:**
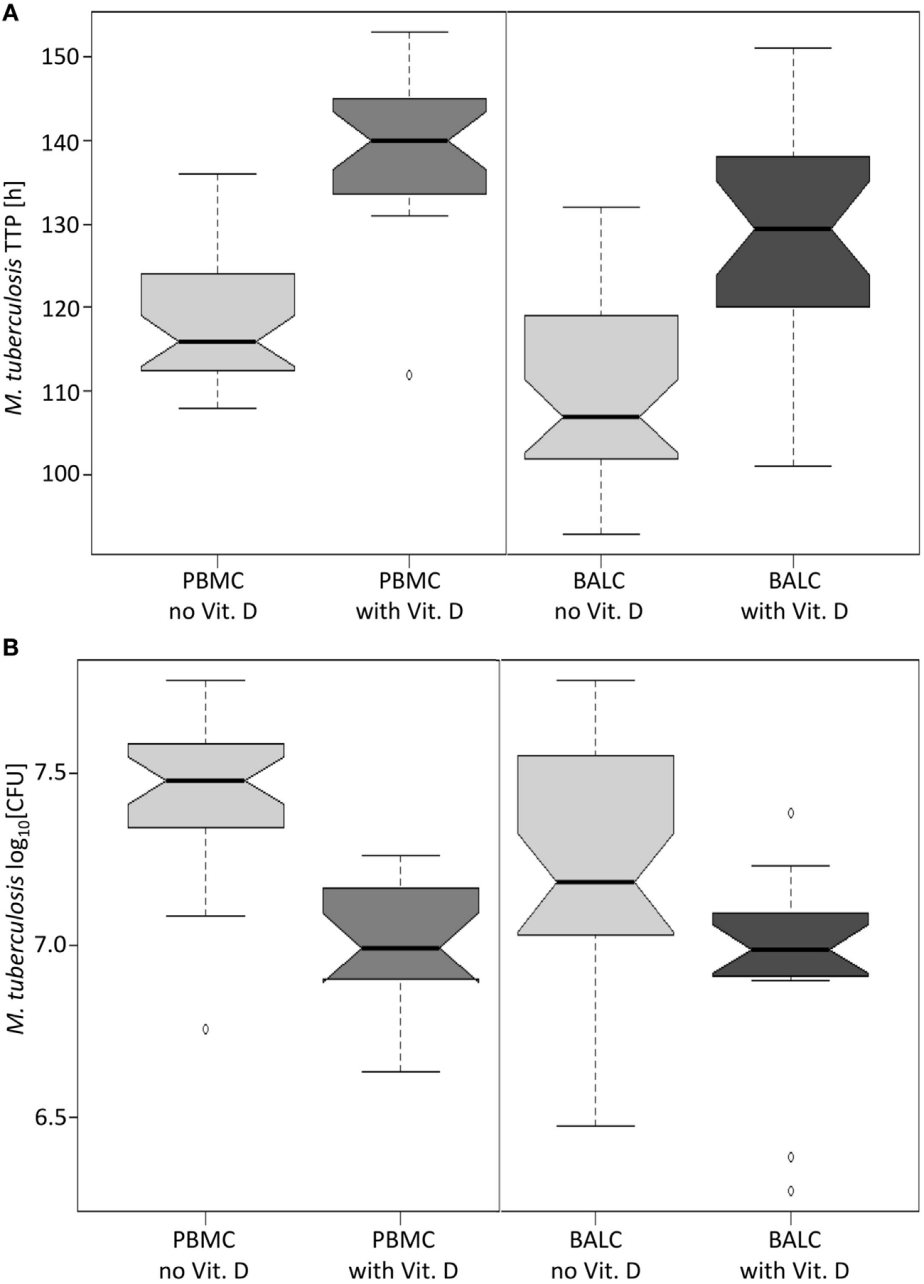
Notched boxplots of the result of vitamin D in peripheral blood mononuclear cells (PBMC) and bronchoalveolar lavage cellss (BALC) of healthy human adults in an *in vitro* infection assay with *Mycobacterium tuberculosis* H37Rv. *M. tuberculosis* growth was measured as **(A)** time to culture positivity (TTP) or **(B)** colony forming units (CFU).

A test series with vitamin D proved that mycobacterial growth control by human immune cells can be measured by the MGIA: in PBMC and BALC, *M. tuberculosis* grew slower in the presence of vitamin D, which is reflected by the estimated 17.2 h higher TTP in PBMC (95% CI: 13.4–21.0 h, *p* < 0.0001) and 20.6 h higher TTP in BALC (95% CI: 16.8–24.3 h, *p* < 0.0001, Figure 6A), as well as in the 0.441 lower log_10_ CFU count in PBMC (95% CI: −0.524 to −0,359, *p* < 0.0001) and 0.226 log_10_ CFU count in BALC (95% CI: −0.348 to −0.184, *p* < 0.0001, Figure 6B).

### Correlation Between IGRA Response and Growth Control in MGIA

**Figure 7:**
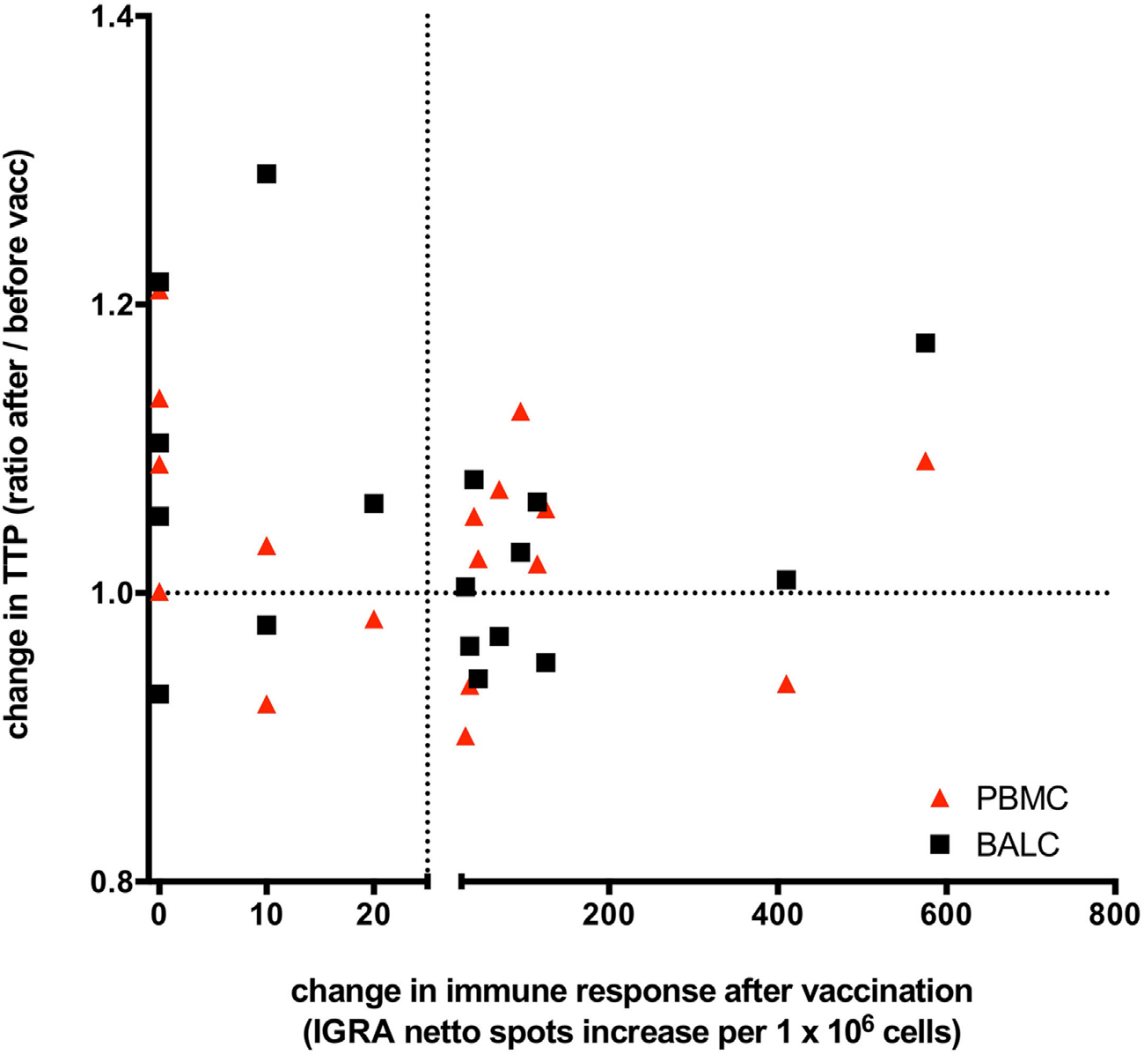
Changes of *Mycobacterium tuberculosis* growth rate plotted against *Mycobacterium bovis* Bacille Calmette–Guérin (BCG) vaccination induced T-cell immune response: the change in time to culture positivity (TTP) in the mycobacterial growth inhibition assay (MGIA) is calculated as TTP ratio after vaccination to before vaccination. The dotted line on the *y*-axis depicts a ratio of 1, indicating the same TTP for *M. tuberculosis*-growth before and after vaccination. A ratio >1 represents a reduced mycobacterial growth (increase in TTP in the second MGIA after BCG-vaccination) and a ratio <1 represents an increased mycobacterial growth (decrease in TTP in the second MGIA after BCG vaccination). TTP in bronchoalveolar lavage cells (BALC) is given in black squares, TTP in peripheral blood mononuclear cells (PBMC) in red triangles. Before vaccination, all participants had a negative interferon-γ release assay (IGRA) enzyme-linked immunospot (ELISpot) response to purified protein derivate from *M. bovis* BCG. On the *x*-axis, a change in the immune response before and after *M. bovis* BCG-vaccination is given as increase of netto spots per 1 million PBMC in the IGRA-ELISpot 8 weeks after vaccination. The dotted line depicts an increase of 25 netto spots per 1 million PBMC. An increase of less than 25 netto spots in the IGRA means that the immune response after BCG-vaccination stayed negative in that individual, whereas BCG-vaccination had induced an IGRA-response in these individuals, who had an increase >25 netto SFC in the second IGRA. Patients presented in the upper right quadrant have gained a successful innate immune response following vaccination (better growth control of *M. tuberculosis* and a new positive cytokine immune response), whereas patients situated in the right lower quadrant are having a new positive cytokine response but miss an enhanced growth control. The upper left quadrant shows better growth control without any adaptive immune response. This overall spread depicts the lack of correlation of immune responses and growth control measured by the MGIA.

To answer the key question of whether growth control after vaccination was influenced by T-cell response, *M. tuberculosis*-growth (TTP) was stratified by IGRA-response. No correlation between the changes of TTP before and after *M. bovis* BCG-vaccination and the induced IGRA T-cell immune response was found (correlation = 0.468, 95% CI: −0.016 to 0.775). *M. bovis* BCG-vaccination-induced IGRA response did not predict the direction of possible changes of any effect on *M. tuberculosis*-growth control in PBMC nor BALC (Figure 7).

### FACS Analysis

Phenotype characterization (Table 2) revealed a significant lower percentage of lymphocytes in BALC than in PBMC (2.06 vs. 43.90% before vaccination and 0.80 vs. 41.50% after vaccination, *p* < 0.0001 for difference between cell-types, *p* = 0.4190 for difference between time points) with a slightly lower CD4/CD8-quotient in BALC than in PBMC (1.66 vs. 3.44% before vaccination and 1.77 vs. 2.52% after vaccination, *p* = 0.0045 for difference between cell-types, *p* = 0.3273 for difference between time points).

**Table 2:** Phenotypical characterization of bronchoalveolar lavage cells (BALC) and peripheral blood mononuclear cells (PBMCs) before and after *Mycobacterium bovis* Bacille Calmette–Guérin (BCG) vaccination by FACS analysis.

Comparison of lung and blood compartment	Before vaccination		After vaccination		*p*-Value for difference between cell-types	*p*-Value for difference between time points
	BALC *n* = 10	PBMC *n* = 12	BALC *n* = 12	PBMC *n* = 12		
CD3+ lymphocytes (% of all cells)	2.06 (0.65–3.23)	43.90 (40.02–48.14)	0.80 (0.51–2.43)	41.50 (29.66–46.14)	<0.0001	0.419
CD4/CD8 quotient (of CD3+)	1.66 (1.30–2.20)	3.44 (2.23–4.33)	1.77 (0.94–2.44)	2.52 (1.72–3.90)	0.0045	0.3273
Perforin+ (in % of CD3+CD4+)	0.15 (0.08–0.36)	0.01 (0.01–0.02)	0.10 (0.03–0.21)	0.02 (0.00–0.03)	0.6217	0.9632
Granzyme B+ (in % of CD3+CD4+)	0.10 (0.06–0.30)	0.08 (0.06–0.15)	0.18 (0.09–0.34)	0.23 (0.09–0.38)	0.6399	0.0506
Granulysin+ (in % of CD3+CD4+)	0.00 (0.00–0.00)	0.00 (0.00–0.00)	0.00 (0.00–0.00)	0.00 (0.00–0.01)	n.a.	0.4691
Perforin+ (in % of CD3+CD8+)	0.11 (0.06–0.26)	0.80 (0.39–1.15)	0.04 (0.01–0.11)	0.71 (0.21–1.50)	0.0026	0.883
Granzyme B+ (in % of CD3+CD8+)	1.16 (0.65–1.33)	2.35 (1.67–3.52)	2.10 (1.59–2.87)	3.70 (2.11–5.04)	0.0847	0.0507
Granulysin+ (in % of CD3+CD8+)	0.04 (0.03–0.07)	0.01 (0.00–0.03)	0.08 (0.04–5.62)	0.03 (0.00–4.64)	0.8058	0.106

*Bronchoalveolar lavage cells and PBMCs were characterized for their surface marker expression of CD3, CD4, CD8, Perforin, Granzyme B, and Granulysin before and after *M. bovis* BCG-vaccination. n.a., not applicable. This table shows the median (IQR) for the specific outcomes stratified by cell-type and time point, and the *p*-values for testing equality between the cell groups and between before and after time points simultaneously*.

Less CD8+ T-cells expressed Perforin in BALC than in PBMC (0.11 vs. 0.80% before vaccination and 0.04 vs. 0.71% after vaccination, *p* = 0.0026 for difference between cell-types, *p* = 0.8830 for difference between time points). The expression of Granzyme B was almost affected by vaccination: granzyme B on CD8+ T-cells was elevated from 1.16 to 2.10% on CD8 + BALC and from 2.35 to 3.70% on CD8 + PBMC (*p* = 0.0507). The expression of Perforin and Granzyme B on CD4+ T-cells was so low, that we would not overestimate any effects seen, the expression of Granulysin on CD4+ T-cells was not detectable.

## Discussion

We evaluated the performance of the MGIA as a marker for immune protection following *M. bovis* BCG-vaccination of healthy adult volunteers. *M. bovis* BCG-vaccination did not have a significant impact on the mycobacterial growth rate in BALC or PBMC and *M. bovis* BCG*-*induced T-cell immune response did not correlate with the mycobacterial growth rate in pulmonary or systemic mononuclear cells.

Due to the absence of an effective vaccine, *M. bovis* BCG is still applied in new-borns from high-incidence countries to reduce the severity of disease (4, 34). Although a promising vaccine candidate MVA85A induced T-cell mediated immune responses, these signals failed to correlate with protective immunity in a clinical trial (12). In the present study, 58.8% of the participants developed a conversion of their PPD-specific IGRA-ELISpot immune response in blood following *M. bovis* BCG-vaccination. Nevertheless, *M. bovis* BCG-vaccination did not result in improved immune control in the *M. tuberculosis* infection assays (neither TTP nor CFU), suggesting that *M. bovis* BCG-vaccination does not improve *M. tuberculosis* growth control. This finding cannot be explained by a failing MGIA but indicates a lack of protective immunity following *M. bovis* BCG vaccination (4, 5). The missing immune response after *M. bovis* BCG-vaccination fits to epidemiological data, showing that *M. bovis* BCG-vaccination does not protect from tuberculosis infection and can only reduce the rates of miliary tuberculosis and tuberculosis meningitis in children (4, 35).

Our results support previous findings that a positive vaccine-antigen-specific IGRA immune response does not correlate with functional immune control for mycobacterial infection *in vivo* and is, therefore, an inappropriate marker to predict immune protection (12).

The MGIA was presented as a promising surrogate for immune protection in previous studies (16, 18). Vitamin D_3_ [1α,25(OH)_2_-Cholecalciferol] is known for its immune-modulating effects on *M. tuberculosis*-control (36–38) and for inducing an antimicrobial activity of human monocytes/macrophages toward *M. tuberculosis*. In a vulnerable population with genetic variations in the vitamin D pathway, an adjuvant treatment with high-dose vitamin D was shown to accelerate the time to sputum culture conversion (39). Possible cellular mechanisms were observed to be associated with expression of cathelicidin (40). In our study, this vitamin D-effect is used as a model to monitor improved immune control as expected in an effective vaccine. In the presence of vitamin D, mycobacterial growth rates decreased (measured as increase in TTP or lower CFU counts) confirming the functionality of the MGIA. Our findings corroborate previous studies with regard to the antimycobacterial activity of vitamin D (38, 41).

Our findings differ from previously published results, where *M. bovis* BCG-vaccination was associated with improved mycobacterial growth control in humans (15, 16). We avoided the potent antituberculosis drug streptomycin and used freshly isolated BALC or PBMC instead of cryopreserved human PBMC or whole blood, third, in our setting, the infection was performed with *M. tuberculosis* and not with *M. bovis* BCG. So far, *M. tuberculosis* has only been used in murine splenocytes (17–19) or murine bone-marrow-derived macrophages (20) to investigate vaccine-induced changes of immune control against *M. tuberculosis*. With regard to vaccine-induced growth inhibition in human cells, the MGIA was successfully performed with *M. bovis* BCG in either PBMC and/or whole blood (15, 16, 22). To our knowledge, the current study represents the first description of the MGIA as a surrogate marker for vaccine-induced antimycobacterial activity using *M. tuberculosis* in human cells (in PBMC and especially in human lung cells), an aspect that has not been addressed so far.

Compared to the mentioned previous studies, which have used MOIs between 0.0001 and 0.0002, it needs to be stated that the MOI of 0.058 used in the current study is significantly higher. In order to make sure that this MOI would allow the measurement of an enhanced antituberculosis response, we included vitamin D to our experiments. As we observed a vitamin D-dependent average TTP increase by 17.2 h in PBMC and 20.6 h in BALC (Figure 6A), we concluded that even at an MOI of 0.058, a given vaccination-dependent antituberculosis directed response leading to prolonged TTP values should have been detectable. This was not the case. Nevertheless, we cannot exclude, that different experimental conditions such as the use of a lower MOI could have led to different results. The current study may, however, indicate that the type of bacteria used in the MGIA needs to be considered. Several authors have shown that *M. bovis* BCG-vaccination of human individuals induces growth inhibition of *M. bovis BCG*, whereas our experimental data suggest that *M. bovis* BCG-vaccination does not lead to growth control of *M. tuberculosis* by human PBMC and BALC.

The TTP in BALC was significantly lower than TTP in PBMC, implicating higher growth rate in BALC. This effect was not seen in CFU read-outs, which may indicate that CFU and TTP measure different conditions. The MGIT-device detects fluorescence, if actively respiring micro-organisms consume the oxygen. In comparison, CFU documents mycobacteria in colonies on agar plates, where *M. tuberculosis* grows typically eugone in firmly attached, beige, dry crumbles, but may differ in colony size. Latter observation may indicate a substantially reduced replication status of a given tiny/small colony. Microbiologists have described these small cell populations as persisters or dormant, non-growing bacteria with the capacity to regrow or viable but not-culturable (42). Regarding to *M. tuberculosis*, it was shown that the replication rate is reduced in small persister-type *M. tuberculosis* in comparison to a population of larger, culture-viable mycobacteria (43). *In vivo* replicating and dormant bacterial populations exist within the same patient (43–45). We assume that small, slow-replicating colonies will be counted in the CFU analysis, but may be left underestimated in the MGIA assay, which documents rates of metabolism and growth by oxygen consumption and explain the observed differences between the TTP and CFU analysis.

Another reason to explain this difference in growth behavior might be cell differences between the lung and the blood compartment (46). Lavage consisted of significantly less lymphocytes than PBMC and additionally a lower number of cytotoxic CD8 + T-cells expressing Perforin was found in BALC in comparison with PBMC. These results once more demonstrate that *in vitro* experiments with PBMC may not imitate the cell-based immune responses and control in human TB (47–49).

Our study is limited by the number of participants and by the lack of an alternative and more promising vaccine candidate for the immune intervention. Although other candidate markers such as mRNA transcripts (50, 51) or cytokines (52) may also appear promising as correlates for protection from *M. tuberculosis* infection, our findings may serve as valuable proof-of-concept for future vaccine evaluations.

In conclusion, vaccination with *M. bovis* BCG did not result in *ex vivo* growth inhibition of *M. tuberculosis* in human alveolar macrophages from healthy volunteers despite the development of antimycobacterial immune responses in peripheral blood.

## Ethics Statement

This study was carried out in accordance with the recommendations of the Declaration of Helsinki (2008) and the Ethics Committee of the University of Lübeck. The protocol was approved by the Ethics Committee of the University Lübeck (14-091). All subjects gave written informed consent in accordance with the Declaration of Helsinki.

## Author Contributions

Conceived and designed the study: JH, CL, and BK. Performed the experiments: JR, ER, and BK. Analyzed the data: JR, LM, PSC, NR, and BK. Wrote the manuscript: JR, JH, LM, PSC, NR, ER, CL, and BK.

## Conflict of Interest Statement

The authors declare that the research was conducted in the absence of any commercial or financial relationships that could be construed as a potential conflict of interest.

## Supplementary Material

Figure S1 In our pre-experiments, the optimal dosage multiplicity of infection was determined. One million peripheral blood mononuclear cells of three different healthy donors were infected with increasing concentrations [colony forming units (CFU)] of the inoculum of *Mycobacterium tuberculosis* H37Rv. The concentration of CFU/mL is plotted against the correlating time to culture positivity (TTP) in *Mycobacterium* growth indicator tubes (MGIT) given in hours (h).

Figure S2 The gating strategy used to identify CD4+ and CD8+ T-cells from peripheral blood mononuclear cells (PBMC) and bronchoalveolar lavage cells (BALCs) is shown in representative density plots from a single person. From left to right, lymphocytes were selected using forward scatter/side scatter (FSC-A/SSC-A)-area, subsequently followed by gating on lymphocyte-singlets (SSC-H/SSC-A), thereafter selecting CD3+ T-cells and stratifying for CD4+ and CD8+ T-cells.
