# Supplementary figures and images for: Mycobacterium Growth Inhibition Assay of Human Alveolar Macrophages as a Correlate of Immune Protection Following Mycobacterium bovis Bacille Calmette–Guérin Vaccination
Source: Front Immunol. 2018 Jul 24;9:1708. doi: 10.3389/fimmu.2018.01708 (PMC6066571; doi:10.3389/fimmu.2018.01708)

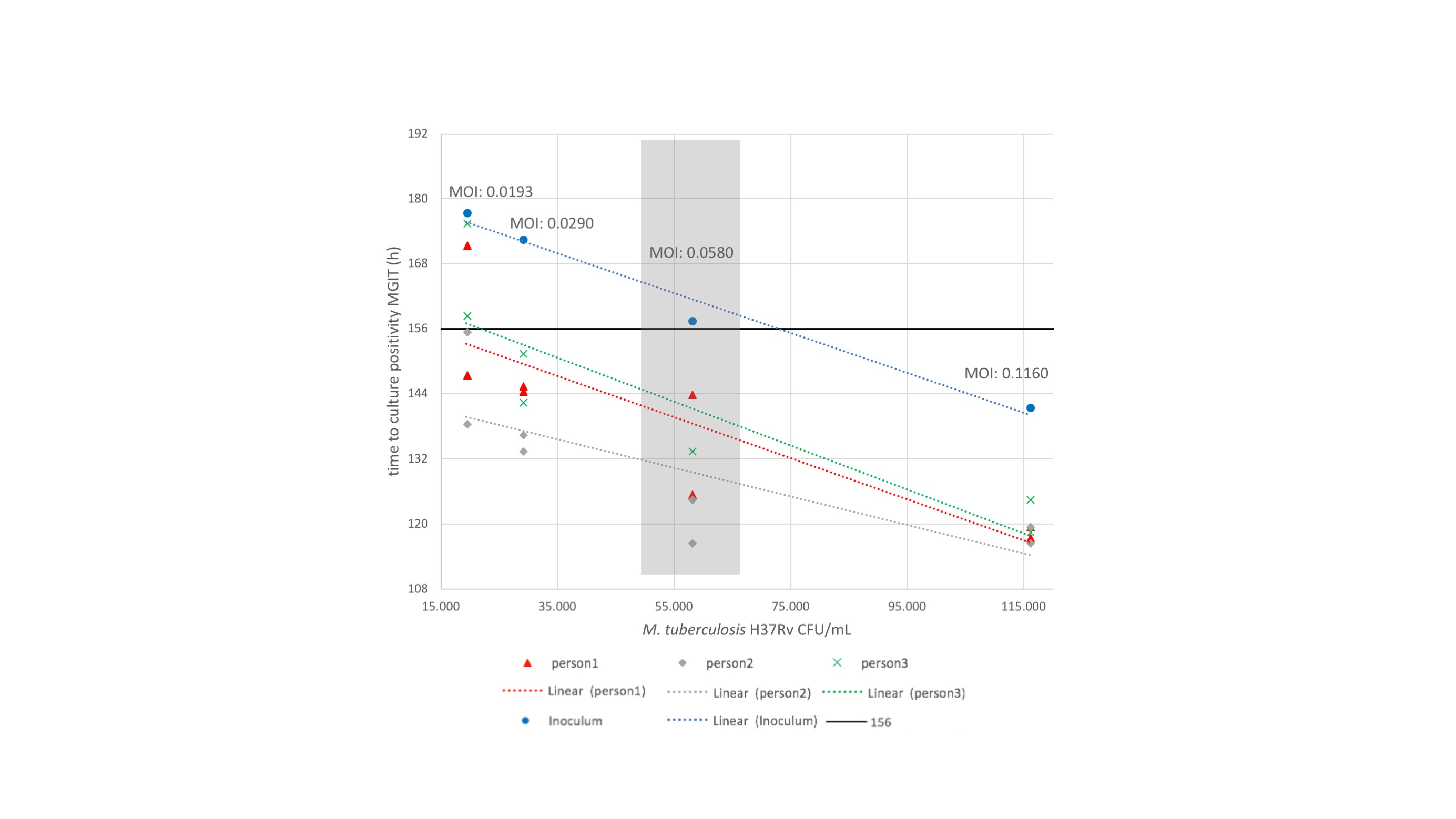

Supplement: Figure S1 — In our pre-experiments, the optimal dosage multiplicity of infection was determined. One million peripheral blood mononuclear cells of three different healthy donors were infected with increasing concentrations [colony forming units (CFU)] of the inoculum of Mycobacterium tuberculosis H37Rv. The concentration of CFU/mL is plotted against the correlating time to culture positivity (TTP) in Mycobacterium growth indicator tubes (MGIT) given in hours (h). [file Image_1.JPEG]

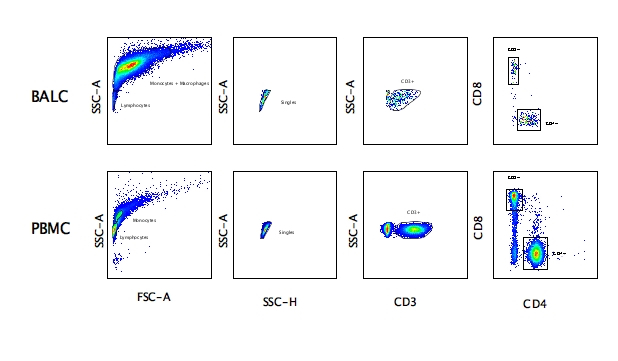

Supplement: Figure S2 — The gating strategy used to identify CD4+ and CD8+ T-cells from peripheral blood mononuclear cells (PBMC) and bronchoalveolar lavage cells (BALCs) is shown in representative density plots from a single person. From left to right, lymphocytes were selected using forward scatter/side scatter (FSC-A/SSC-A)-area, subsequently followed by gating on lymphocyte-singlets (SSC-H/SSC-A), thereafter selecting CD3+ T-cells and stratifying for CD4+ and CD8+ T-cells. [file Image_2.JPEG]
